# Beneficial effects of prehospital versus immediate in-hospital blood products during resuscitation in two models of severe military injury

**DOI:** 10.1186/cc14429

**Published:** 2015-03-16

**Authors:** S Watts, G Nordmann, C Wilson, A Carter, H Poon, E Kirkman

**Affiliations:** 1 Dstl, Salisbury, UK

## Introduction

Acute trauma coagulopathy (ATC) is seen in 30 to 40% of severely injured trauma casualties. Early use of blood products is thought to attenuate ATC. This study determined the potential impact of prehospital versus immediate in-hospital packed red blood cells and fresh frozen plasma (PRBC:FFP) in two models of severe battlefield injury.

## Methods

This is a prospective randomised controlled trial using *in vivo *models of injury conducted in accordance with the Animals (Scientific Procedures) Act, 1986. Two injury strands were investigated in 43 terminally anaesthetised Large White pigs: whole body blast exposure (Bl) or no blast (ShBl) plus soft tissue injury and haemorrhage. Thirty minutes later animals were randomly allocated to a 60-minute simulated prehospital hypotensive resuscitation with either PRBC:FFP (1:1 ratio) or 0.9% saline (Early and Late groups respectively). This was followed by 150 minutes of simulated in-hospital resuscitation with a revised normotensive target whereby PRBC:FFP was initiated in the Late group and continued in the Early group.

## Results

In the ShBl injury strand there was a significant reduction in ATC in Early compared with Late PRBC:FFP treatment (TEG R and K times) in both the prehospital (*P *= 0.004 and *P *= 0.003 respectively, ANOVA) and early in-hospital (*P *= 0.002 and *P *= 0.005) phases, although clotting was normalised in the Late group within 60 minutes of initiating PRBC:FFP. Prehospital base deficit (BD) was significantly attenuated in ShBl Early versus Late (9.0 ± 2.1 vs. 14.4 ± 2.2 mM). BD improved in both Early and Late treatment groups during the in-hospital phase but remained greater in the Late group throughout (*P *< 0.001). In the Bl injury strand the trend in coagulation was similar to that seen in the ShBl injury strand (but the differences between Early and Late did not attain statistical significance). By contrast, Early versus Late PRBC:FFP treatment did not result in a difference in BD in the Bl strand. Finally, there was no difference in the total amount of PRBC:FFP used between the two treatments in either injury strand, but in both injury strands the Early treatment groups required significantly less saline (*P *< 0.001).

## Conclusion

Prehospital use of PRBC:FFP may attenuate ATC and improve physiological status. Furthermore the amount of crystalloid may be reduced with potential benefit of reducing the third-space effect and later tissue oedema.

## Acknowledgements

© Crown copyright 2014. Published with the permission of the Dstl on behalf of the Controller of HMSO.

